# Vital Sign Detection during Large-Scale and Fast Body Movements Based on an Adaptive Noise Cancellation Algorithm Using a Single Doppler Radar Sensor

**DOI:** 10.3390/s20154183

**Published:** 2020-07-28

**Authors:** Zi-Kai Yang, Heping Shi, Sheng Zhao, Xiang-Dong Huang

**Affiliations:** 1 School of Microelectronics, Tianjin University, Tianjin 300072, China; zikai_yang@tju.edu.cn (Z.-K.Y.); shengzhao@tju.edu.cn (S.Z.); 2 School of Automobile and Transportation, Tianjin University of Technology and Education, Tianjin 300222, China; 3 School of Electronic Information Engineering, Tianjin University, Tianjin 300072, China; xdhuang@tju.edu.cn

**Keywords:** continuous-wave Doppler radar, random body movement, vital sign detection, adaptive noise cancellation, new-type discrete cosine transform

## Abstract

The non-contact detection of human vital signs (i.e., respiration rate (RR) and heartbeat rate (HR)) using a continuous-wave (CW) Doppler radar sensor has great potential for intensive care monitoring, home healthcare, etc. However, large-scale and fast random body movement (RBM) has been a bottleneck for vital sign detection using a single CW Doppler radar. To break this dilemma, this study proposed a scheme combining adaptive noise cancellation (ANC) with polynomial fitting, which could retrieve the weak components of both respiration and heartbeat signals that were submerged under serious RBM interference. In addition, the new-type discrete cosine transform (N-DCT) was introduced to improve the detection accuracy. This scheme was first verified using a numerical simulation. Then, experiments utilizing a 10-GHz Doppler radar sensor that was built from general-purpose radio frequency (RF) and communication instruments were also carried out. No extra RF/microwave components and modules were needed, and neither was a printed circuit board nor an integrated-chip design required. The experimental results showed that both the RR and HR could still be extracted during large-scale and fast body movements using only a single Doppler radar sensor because the RBM noises could be greatly eliminated by utilizing the proposed ANC algorithm.

## 1. Introduction

Phase-modulation microwave Doppler radar sensors [1], which are attractive due to the advantages of being contactless and able to penetrate obstacles [2], have gained increasing attention in the field of monitoring vital signs, i.e., respiration rate (RR) and heartbeat rate (HR) [3,4,5,6,7,8]. The Doppler radar sensor transmits a single-tone CW signal to a subject and receives the reflected signals, which carry the information of the physiological movements of the subject in its phase. In the receiver of the sensor, the information regarding the RR and HR of the subject can be extracted by demodulating the received signals. This technique enables short-distance vital sign detection of humans without contacting the testers. With the non-contact vital sign detection capability, the CW Doppler radar sensor has great potential for intensive care monitoring [9], home healthcare, and long-term monitoring applications [10].

Although great theoretical and technological efforts have been made in the past few decades [11,12,13,14,15], such as the use of machine learning algorithms to improve the detection accuracy of HR [15], this detection method still has not been widely applied. This is mainly caused by a bottleneck where weak vital signs tend to be submerged by large-scale and fast random body movements (RBMs) [16,17,18,19,20,21,22]. Specifically, the displacement during an RBM is at the level of tens of centimeters, compared to that of the chest wall movement arising from respiration and heartbeat, which is only at the level of several millimeters [21]. As a result, the RBM is a substantial noise source that can destroy the vital sign signals and significantly reduce the detection accuracy [22]. Therefore, to make the detection method more practical and widely applicable, the key problem is how to remove the influence of RBMs.

To overcome the RBM effect, significant effort has been dedicated over the past few decades. In References [16,17,18,19,20], to achieve both RR and HR detection, the RBM was removed using two Doppler radar systems or a single Doppler radar system with extra hardware, e.g., a camera [18]. However, these methods suffer from limitations, such as the antenna/camera alignments and inconvenient usage in practical environments. In addition, large-scale or fast RBM cannot be removed using these methods [21]. To overcome these defects, Lv, et al. [21] proposed a matched filter algorithm to remove large-scale RBM using a single Doppler radar system without extra hardware. However, this method is only effective on the condition that there exists a period during which the velocity of the RBM must be close to 0 mm/s. This may limit its extensive use. In Tu, et al. [22], a single Doppler radar system was built to manage the effect of the body movement velocity, yet it still had difficulty in detecting the HR. In summary, using a single Doppler radar to extract both RR and HR during large-scale and fast RBMs is the key to making the detection technique widely applicable.

In this study, to remove the large-scale and fast RBMs with only a single Doppler radar sensor, a scheme combining adaptive noise cancellation (ANC) with polynomial fitting was proposed to retrieve the weak components of the respiration and heartbeat submerged in serious RBM interference. Furthermore, a new-type discrete cosine transform (N-DCT)-based spectrum extractor was introduced to achieve the concurrent detection of the RR and HR. In addition, general-purpose radio frequency (RF) and communication instruments were used to build a Doppler radar system. For the instrument-based radar sensor, no extra RF/microwave components and modules were needed, and neither was a printed circuit board or an integrated-chip design. Both simulations and experimental tests were conducted to verify the performance of the proposed techniques. Furthermore, the measurement results on human subjects are reported and discussed.

## 2. RBM Cancellation

### 2.1. The Effect of Random Body Movements

**Figure 1:**
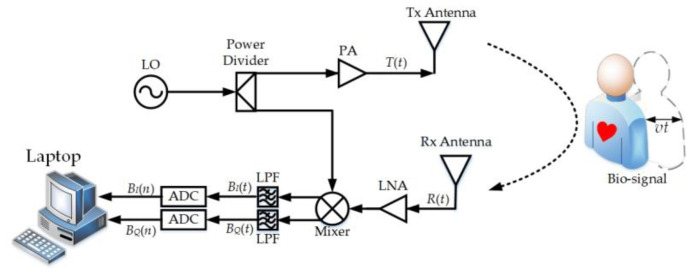
The block diagram of the random body movement (RBM) modulation effect and the Doppler radar sensor. ADC: Analog-to-Digital Converter, LO: Local Oscillator, LNA: Low Noise Amplifier, LPF: Low-Pass Filter, PA: Power Amplifier, Rx: Receiver, Tx: Transmitter.

Figure 1 shows the block diagram of the RBM modulation effect and the Doppler radar sensor. For a non-contact Doppler radar vital sign detection system, the transmitted single-tone CW signal is modeled as:

(1) S(t)=cos[2πft+ϕ(t)],

where *f* is the transmitted frequency and *ϕ*(*t*) is the total phase noise of the transmitter. Assuming that the subject stands at an initial distance *d*_0_ away from the radar, then, after a round-trip delay, the reflected signal received by the radar is given as:

(2) $$R(t)=\cos[2\pi ft-\frac{4\pi d_{0}}{\lambda }-\frac{4\pi x(t)}{\lambda }+\phi (t-\frac{2d_{0}}{c})],$$

where *λ* is the wavelength of the transmitted signal and *c* is the speed of the signal (the propagation velocity of light). *x*(*t*) is the entire movement, which includes the respiration *x_r_*(*t*), heartbeat *x_h_*(*t*), and body motion *x_b_*(*t*), i.e.,

(3) $$\begin{array}{cc} x(t) & =x_{r}(t)+x_{h}(t)\pm x_{b}(t)\\ & =m_{r}\sin\omega_{r}t+m_{h}\sin\omega_{h}t\pm vt, \end{array}$$

where *m_r_* and *ω_r_* are the magnitude and angular frequency of the respiration, respectively; *m_h_* and *ω_h_* are the magnitude and angular frequency of the heartbeat, respectively; and *v* is the velocity of the body motion, which can be considered constant over a short time window [22]. Furthermore, the “±” sign in Equation (3) represents the body motion directions. Specifically, when the subject is moving away from the Doppler radar (i.e., the distance between the subject and the radar is becoming longer), a plus sign holds, and vice versa. In the receiver, the local oscillator (LO) signal is derived from the same source as the transmitted signal and mixed with the received signal using the quadrature mixer. After the received signal is quadrature down-converted and sampled, the baseband in-phase (*I*) and quadrature-phase (*Q*) outputs can be expressed as:

(4) $$\begin{array}{l} B_{I}(n)=\cos\left[\frac{4\pi d_{0}}{\lambda }+\frac{4\pi x_{r}(n)}{\lambda }+\frac{4\pi x_{h}(n)}{\lambda }\pm \frac{4\pi x_{b}(n)}{\lambda }+\phi \right],\\ B_{Q}(n)=\sin\left[\frac{4\pi d_{0}}{\lambda }+\frac{4\pi x_{r}(n)}{\lambda }+\frac{4\pi x_{h}(n)}{\lambda }\pm \frac{4\pi x_{b}(n)}{\lambda }+\phi \right], \end{array}$$

where *ϕ* is the total residual phase, which can be ignored according to the range correlation theory [23]. Then, with the *B_I_*(*n*) and *B_Q_*(*n*), the extended differentiate and cross multiply (DACM) algorithm [24] is applied to extract the phase as follows:

(5) $$\begin{array}{cc} \varphi (n) & =\frac{4\pi d_{0}}{\lambda }+\frac{4\pi x_{r}(n)}{\lambda }+\frac{4\pi x_{h}(n)}{\lambda }\pm \frac{4\pi x_{b}(n)}{\lambda }+\phi \\ & =\sum_{m=2}^{n}\frac{B_{I}(m)[B_{Q}(m)-B_{Q}(m-1)]-[B_{I}(m)-B_{I}(m-1)]B_{Q}(m)}{B_{I}{\left(m\right)}^{2}+B_{Q}{\left(m\right)}^{2}}. \end{array}$$

By combining Equation (3) with Equation (5), the entire movement $\hat{x}$(*n*) = *x_r_*(*n*) + *x_h_*(*n*) ± *x_b_*(*n*) can be expressed as follows:

(6) $$\hat{x}(n)\approx \frac{\varphi (n)\cdot \lambda }{4\pi }-d_{0}.$$

### 2.2. Adaptive Noise Cancellation (ANC)

**Figure 2:**
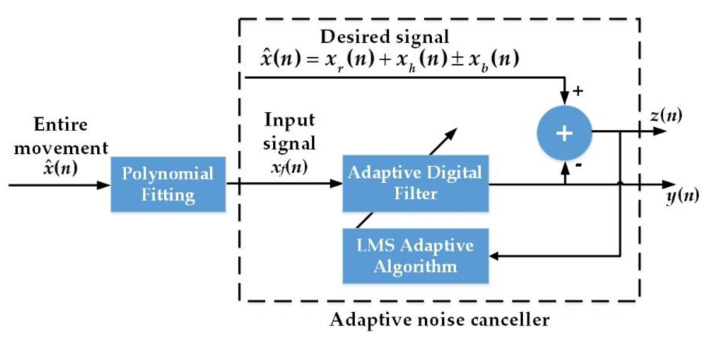
The schematic of the adaptive noise cancellation (ANC). LMS: Least-Mean-Square.

To extract the weak vital sign components (i.e., *x_r_*(*n*) and *x_h_*(*n*)) from $\hat{x}$(*n*), the ANC, which is based on the principles of adaptive filtering and results in optimal noise reduction without distorting the signals, is introduced. The traditional filter is optimal only when the statistical characteristics of the input data match the a priori information on which the design of the filter is based. Different from the traditional filter, ANC relies on a recursive algorithm for its operation, which makes it possible for the filter to perform satisfactorily in an environment where complete knowledge of the relevant signal characteristics is not available [25]. Figure 2 illustrates the schematic of the ANC technique. The demodulated signal $\hat{x}$(*n*), which corresponds to the combination of body movement and vital signs, is treated as the desired signal. Furthermore, another waveform approximating the body motion signal *x_b_*(*n*) needs to be constructed as the ANC’s input signal. To make the constructed waveform as close as possible to the body movement signal *x_b_*(*n*), the method of polynomial fitting is applied to construct the input signal *x_f_*(*n*). The *Y*th order polynomial fitting can be expressed as:

(7) $$x_{f}(n)=\sum_{i=0}^{Y}p_{i}n^{i},$$

where *n^i^* means that the order of the variable *n* is *i* and *p_i_* (*i* = 0, 1, …, *Y*) are the constant coefficients that can minimize the mean square error:

(8) $$\mathrm{MSE}=\frac{1}{N}{\sum_{n=1}^{N}\left[x_{f}(n)-\hat{x}(n)\right]}^{2},$$

where *N* is the number of data points.

After the polynomial fitting is used to construct the input signal *x_f_*(*n*), ANC is applied to obtain another signal *y*(*n*), which is closer to *x_b_*(*n*) than *x_f_*(*n*). Assuming that the estimate of the unknown tap-weight vector of the adaptive digital filter in Figure 2 is **w**(*n*), then the output vector **y**(*n*) (i.e., the vector form of signal *y*(*n*)) can be expressed as:

(9) $$y(n)=x_{f}{\left(n\right)}^{T}w(n)=w{\left(n\right)}^{T}x_{f}(n),$$

where **x***_f_*(*n*) is the input signal vector (i.e., the vector form of signal *x_f_*(*n*)) and *T* denotes transposition. As a result, the estimation error vector **z**(*n*) (i.e., the vector form of signal *z*(*n*)) can be written as:

(10) $$\begin{array}{cc} z(n) & =\hat{x}(n)-y(n)\\ & =\hat{x}(n)-w{\left(n\right)}^{T}x_{f}(n), \end{array}$$

where $\hat{x}$(*n*) is the vector form of signal $\hat{x}$(*n*). The estimation error vector **z**(*n*) is input into the least-mean-square (LMS) adaptive algorithm part in Figure 2. In the LMS adaptive algorithm part, the present value of the estimated weight vector **w**(*n*) is updated as **w**(*n* + 1):

(11) $$w(n+1)=w(n)+2\mu x_{f}(n)z(n),$$

where *µ* is the step-size parameter. The **w**(*n* + 1) is substituted into Equation (9) and replaces **w**(*n*). When the mean square of **z**(*n*) is the minimum, the cycle will end. Therefore, with the ANC employed, the signal *y*(*n*), which is closer to *x_b_*(*n*) than *x_f_*(*n*), is available at the output of the ANC. Hence, after the adaptive filtering converges, the output of the ANC’s subtractor (i.e., *z*(*n*) = $\hat{x}$(*n*) − *y*(*n*)) can be regarded as *x_r_*(*n*) + *x_h_*(*n*), which facilitates further analysis of the RR and HR.

**Figure 3:**
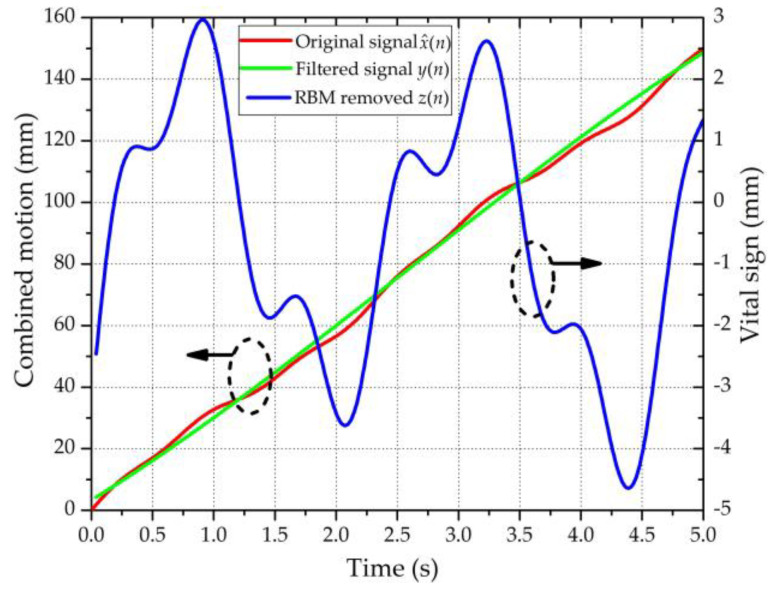
Simulation template processed using the ANC. The green and red curves refer to the left ordinate; the blue curve refers to the right ordinate.

A simulation was conducted to demonstrate the effect of the ANC. In general, most of the respiration was typically within a frequency range of less than 0.6 Hz, whereas the heartbeat lay within a frequency range of 0.8–3 Hz. The body movement caused by respiration was typically less than 1 cm and the body movement caused by the heartbeat was less than 2 mm [25]. In this study, we considered that the respiration was 3 mm in amplitude with a frequency of 0.4 Hz, whereas the heartbeat was 1 mm in amplitude with a frequency of 1.3 Hz. The order of the polynomial fitting was taken to be 3 (see Section 2.3 for further analysis) and the velocity of the body motion was taken to be 30 mm/s. Figure 3 shows the original signal $\hat{x}$(*n*), the filtered signal *y*(*n*), and the difference output *z*(*n*).

### 2.3. New-Type Discrete Cosine Transform.

As described in Section 2.1, the velocity of the body motion can be considered as a constant over a short duration. However, when the time window is decreased, the width of the spectral main-lobe (ML) increases [26]. In other words, as the time window shortens, the spectral peak appears more blurred. As a result, when the vital sign signal (i.e., the RBM-removed signal *z*(*n*)) is analyzed for a short time window, the accuracy of the extracted RR and HR will be reduced.

To highlight the spectral peak to improve the extraction accuracy of the RR and HR, an *M*-point new-type discrete cosine transform (N-DCT) was proposed in Park et al. [27] as follows:

(12) $$X_{DCT}[k]=2\sum_{n=0}^{L-1}z(n)\cos(\frac{2\pi k}{M}n),$$

where *L* is the sample length and *k* refers to the frequency index. As Narasimhan et al. [28] pointed out, the ML of the N-DCT-based spectrum is narrower than that of the commonly-used Fast Fourier Transform (FFT) spectrum. Although the frequency resolution cannot be increased, the zero padding is still applied to reduce the spaced interval in the frequency domain. Both in the simulations and experiments, the sampling frequency *f*_s_ was set to 100 Hz and the sample length *L* was set to 500.

**Figure 4:**
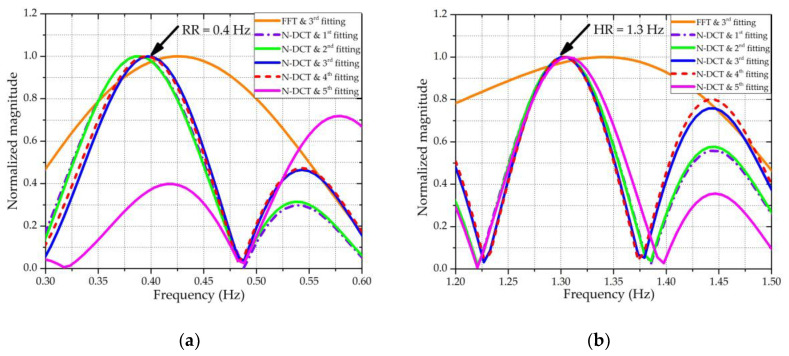
Results after applying the new-type discrete cosine transform (N-DCT) and Fast Fourier Transform (FFT). (**a**) The frequency spectra of respiration, where the reference frequency of the RR was 0.4 Hz. (**b**) The frequency spectra of the heartbeat, where the reference frequency of the HR was 1.3 Hz.

Other than detecting the spectral components included in *z*(*n*), the N-DCT can also be used to evaluate which polynomial fitting order is most appropriate for the input signal *x_f_*(*n*) in Section 2.2. Using the same simulation conditions as in Section 2.2, Figure 4 shows the comparison of the extracted results using N-DCT and traditional FFT. In addition, the extracted results with different polynomial fitting orders used for the ANC are also compared. In Figure 4, the “FFT & 3rd fitting” means that the vital sign signals were extracted based on the third-order polynomial fitting and analyzed using FFT, and so on. It should be noted that the spectra of the heartbeat were obtained after the *z*(*n*) signals were high-pass filtered with *f*_stop_ = 0.7 Hz and *f*_pass_ = 0.9 Hz to eliminate the interference arising from respiration. By observing the position of the spectral peak, the results of the RR obtained using the third- and fourth-order polynomial fittings were more accurate than those obtained using the first-, second-, and fifth-order polynomial fittings. Specifically, as shown in Figure 4a, among the five cases of different orders, the peak positions of the third- and fourth-order polynomial fittings almost exactly fell at the ideal RR of 0.4 Hz. For ease of calculation, the third-order polynomial fitting was selected to construct the input signal *x_f_*(*n*) in Section 2.2. To compare the performance of FFT and N-DCT under the same conditions, the use of the FFT method to extract vital signs also needed to be based on the third-order polynomial fitting. From both Figure 4a,b, it can be seen that the extracted results through N-DCT were more accurate than an FFT for both the RR and HR under the third-order polynomial fitting.

**Figure 5:**
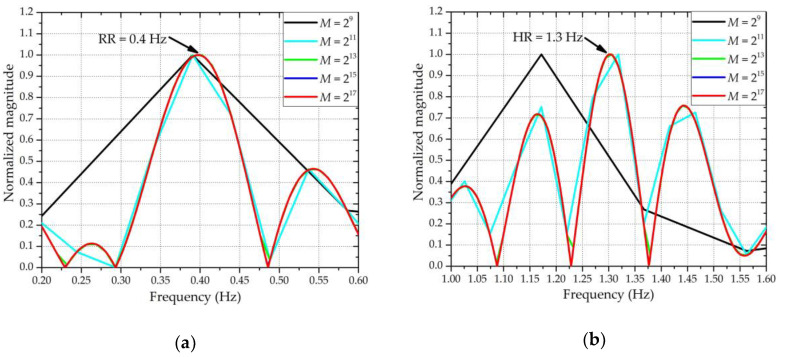
Results with different values of *M*. (**a**) The frequency spectra of respiration, where the reference frequency of the RR was 0.4 Hz. (**b**) The frequency spectra of the heartbeat, where the reference frequency of the HR was 1.3 Hz.

To show whether the value of *M* in Equation (12) affected the extracted accuracy of the RR and HR, Figure 5 shows the extracted vital sign results with different values of *M* using the N-DCT under the third-order polynomial fitting. It can be seen from Figure 5 that when the value of *M* was too small, i.e., *M ≤* 2^11^, the accuracy of the extracted results was degraded. However, when *M* was large enough, i.e., *M ≥* 2^13^, the accuracy of the extracted results was not significantly improved. To ensure that the extraction results of the vital signs were not affected by the value of *M*, *M* was set to 2^17^.

## 3. Experiments

**Figure 6:**
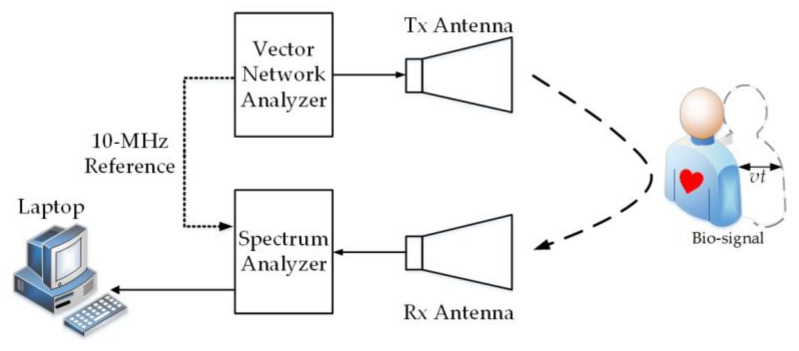
Measurement setup for the instrument-based Doppler radar sensor.

To build the Doppler radar sensor quickly, an instrument-based Doppler radar sensor was realized by utilizing general-purpose RF and communication instruments that are widely used and equipped in RF/microwave laboratories. Figure 6 shows the measurement setup of the radar sensor, which was straightforward to build by connecting instruments. It can be seen that no extra RF/microwave components and modules were needed, nor did it require a printed circuit board or an integrated-chip design. The sensor included a vector network analyzer (N5242A PNA-X, Agilent, Santa Clara, US), a spectral analyzer (R&S FSV30, Rohde & Schwarz, Muenchen, Germany), and two antennas (RB-84SGAH20, Talent Microwave, Suzhou, China). The operating frequency range of this antenna was 6.57–10 GHz. To achieve the highest detection sensitivity, the operating frequency of 10 GHz was selected. To make all the components in the transceiver synchronized and achieve coherent demodulation, a 10-MHz reference signal generated from the vector network analyzer was used as the clock for the whole system.

**Figure 7:**
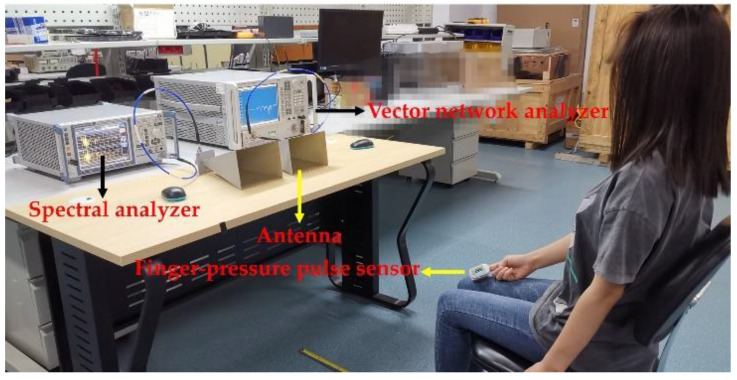
Experimental setup for the monitoring of the RR and HR during RBMs.

Figure 7 shows the experimental setup for the monitoring of the RR and HR under large-scale and fast body movements. In the experiment, the vector network analyzer acted as a transmitter to generate a single-tone CW signal with a frequency of 10 GHz and a power of 10 dBm. The reflected signal was received by the antenna and demodulated in the spectral analyzer. During the experiment, the subject was asked to sit at an initial distance of 1.5 m before the Doppler radar sensor and sway back and forth with different amplitudes. For the movement in each direction, the duration was approximately 3–5 s. At the same time as the body moved, the subject tried to keep a normal breathing pattern. To obtain the reference frequency of respiration, the subject was asked to breathe following the rhythm of a metronome. When the rhythm was changed, the body motion direction was changed too. Therefore, the reference frequency of respiration could be calculated according to the rhythm of the metronome. However, we must acknowledge that it was difficult for the subject to follow the metronome’s rhythm perfectly when the movement direction was changed. As a result, there were some small errors in the reference frequency of the respiration. For the heartbeat, a finger pulse oximeter YX303 (Yuwell, Suzhou, China) measurement served as the HR reference. The activation of the radar system was controlled by a mouse. To achieve the synchronization between the radar and the finger pulse oximeter, the subject pressed the switch of the finger pulse oximeter and the mouse simultaneously. Since the synchronization between the radar and the finger pulse oximeter was done manually, it may have also caused some small errors.

## 4. Results and Discussion

**Figure 8:**
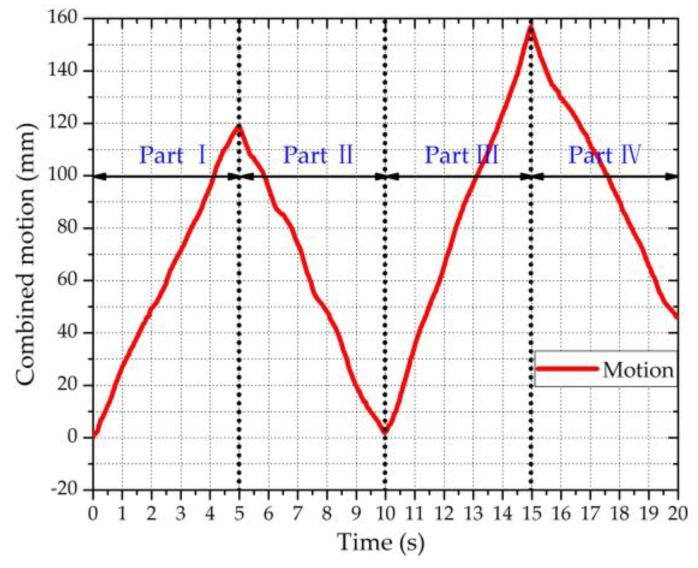
The demodulated combined motion for a duration of 20 s.

After applying the extended DACM algorithm to the demodulated quadrature baseband signals *B_I_*(*n*) and *B_Q_*(*n*), the combined motion $\hat{x}$(*n*) was obtained. Figure 8 shows the combined motion, which consisted of the vital signs and body motion for a duration of 20 s. Based on the movement direction, the combined motion in Figure 8 was divided into four parts (i.e., part Ⅰ, part Ⅱ, part Ⅲ, part Ⅳ). It should be noted that the combined motion displacement in Figure 8 refers to the relative displacement from the initial position. Figure 8 shows that the maximum displacement was about 160 mm. In addition, it can be seen that the vital sign signals were completely submerged by the body movement signals. It should be noted that even though the direction of the body movement relative to the radar was not known, we could also obtain the sign before *x_b_*(*t*) in Equation (3) (i.e., $x(t)=x_{r}(t)+x_{h}(t)\pm x_{b}(t)$) using the demodulated combined motion $\hat{x}$(*n*). Specifically, the sign before *x_b_*(*t*) in Equation (3) is the same as the sign of the speed direction of the demodulated combined motion. As shown in Figure 8 below, because the displacement kept increasing in part Ⅰ and kept decreasing in part Ⅱ, the speed directions in parts Ⅰ and Ⅱ were “+” and “−,” respectively. Therefore, the signs before *x_b_*(*t*) in Equation (3) corresponding to parts Ⅰ and Ⅱ were “+” and “−,” respectively. By analogy, the speed directions in parts Ⅲ and Ⅳ were “+” and “−,” respectively. Therefore, the signs in Equation (3) corresponding to parts Ⅲ and Ⅳ were “+” and “−,” respectively.

**Figure 9:**
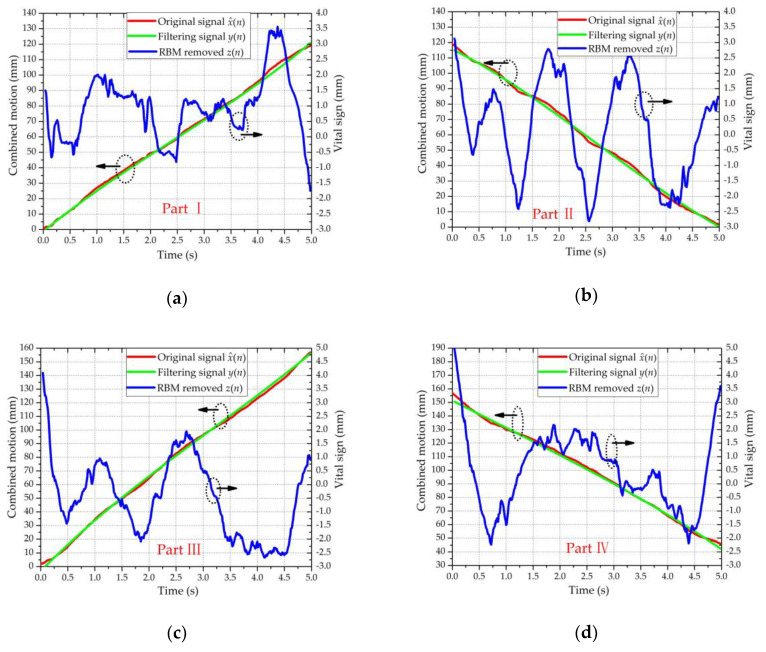
Signals processed with the ANC. The green and red curves refer to the left ordinate; the blue curve refers to the right ordinate: (**a**) part Ⅰ, (**b**) part Ⅱ, (**c**) part Ⅲ, and (**d**) part Ⅳ.

**Figure 10:**
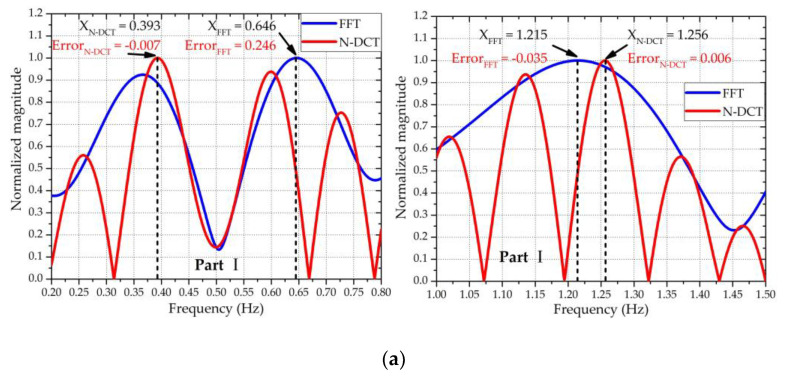

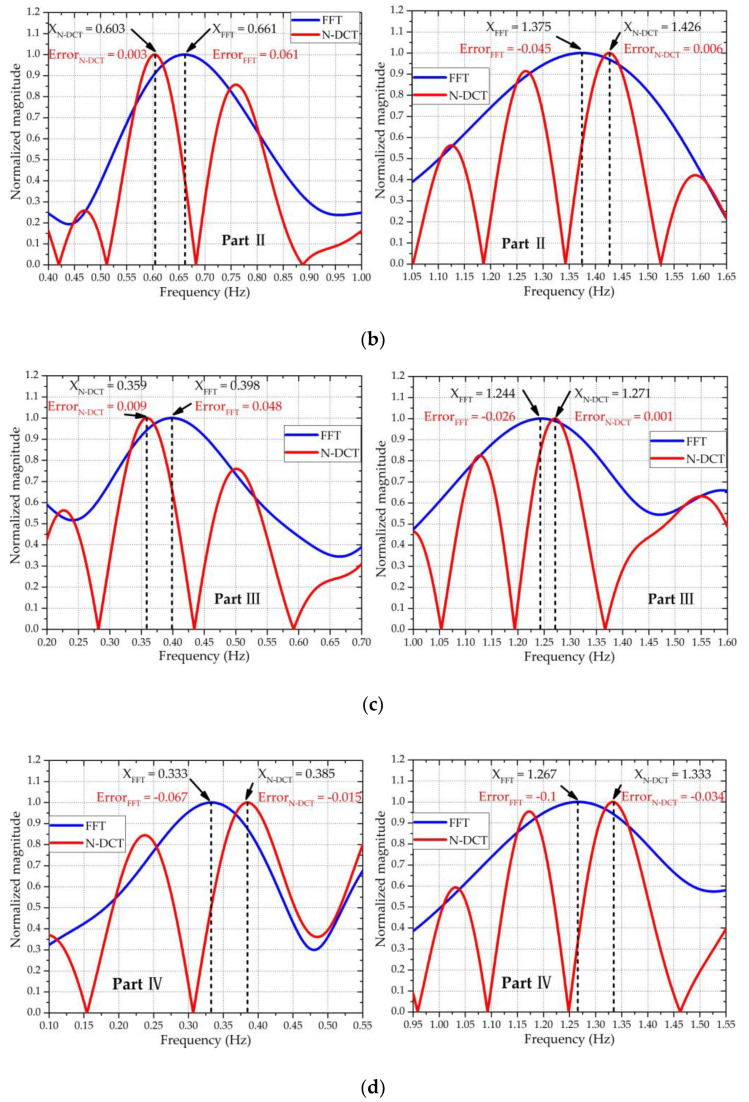
Frequency spectra of the respiration and heartbeat of the four parts corresponding to Figure 9, with the RR in the left figures and the HR in the right figures: (**a**) part Ⅰ—the true RR = 0.4 Hz, HR = 1.25 Hz; (**b**) part Ⅱ—the true RR = 0.6 Hz, HR = 1.42 Hz; (**c**) part Ⅲ—the true RR = 0.35 Hz, HR = 1.27 Hz; and (**d**) part Ⅳ—the true RR = 0.4 Hz, HR = 1.367 Hz.

To extract the vital sign signals (i.e., the RBM-removed signal *z*(*n*)) from the combined motion signals $\hat{x}$(*n*), ANC was utilized to remove the RBM signals *x_b_*(*n*). Figure 9 shows the signal processing results of the four parts in Figure 8, reflecting that the body motions were almost removed by the ANC. Based on the RBM-removed signal *z*(*n*), the RR and HR were extracted using N-DCT and FFT methods with the same 5 s window width. Figure 10 compares the spectra of the respiration and heartbeat resulting from the N-DCT and FFT, respectively.

Figure 10 clearly exhibits that the N-DCT did better at highlighting the main lobes than FFT did for the four different RBMs. Accordingly, the N-DCT yielded higher detection accuracy than an FFT for both the RR and HR. Table 1 summarizes the measurement errors of the RR and HR when using FFT and N-DCT. The error was used to quantify the extracted accuracy of the FFT and N-DCT methods, which was defined as:

(13) $$error=\frac{R_{measure}-R_{ref}}{R_{ref}}\times 100\%,$$

where *R_measure_* is the measured RR or HR and *R_ref_* is the reference rate of the RR or HR. Furthermore, the average error was calculated using:

(14) $$\mathrm{Average}\;\mathrm{error}=\frac{1}{4}\sum_{l=1}^{4}\left|error_{l}\right|.$$

As can be seen from Table 1, compared with the traditional FFT method using a 5 s time window, the N-DCT reduced the average error of the RR from 25.53% to 2.14% and reduced the average error of the HR from 3.84% to 0.87%.

**Figure 11:**
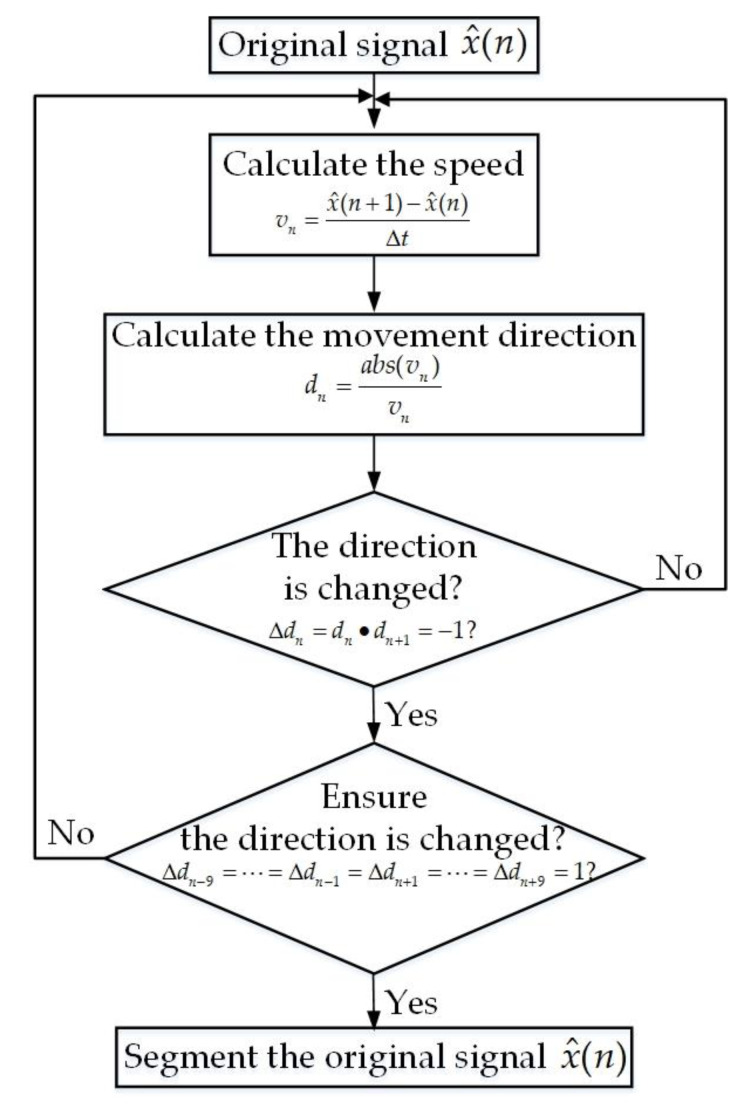
The flowchart of the algorithm used to segment the body displacement signals.

From Table 1, it can be obtained that the vital signs were extracted during large-scale and fast body movements based on the method proposed in this study using only one single Doppler radar sensor. However, in a real scenario, random body movements could include irregular back and forth movements or a single-direction movement with a speed variation. To manage a real scenario, we can divide a long time window of a vital sign signal into several short time windows, where each short time window contains the body movement toward one direction with a constant speed such that the method proposed in this study can still be used. Therefore, to verify the proposed method can be applied in the real-world scenario, the length of the time window needed to be shortened and an algorithm was needed to segment the body displacement signals when the body movement direction changed. According to Tu et al. [22], a window length of 3–5 s can be considered a short time window. As a result, it must be verified whether the vital signs can be extracted under a 3 s time window. In addition, to segment the body displacement signals based on the body movement direction, Figure 11 shows the flowchart of the segmentation algorithm.

**Figure 12:**
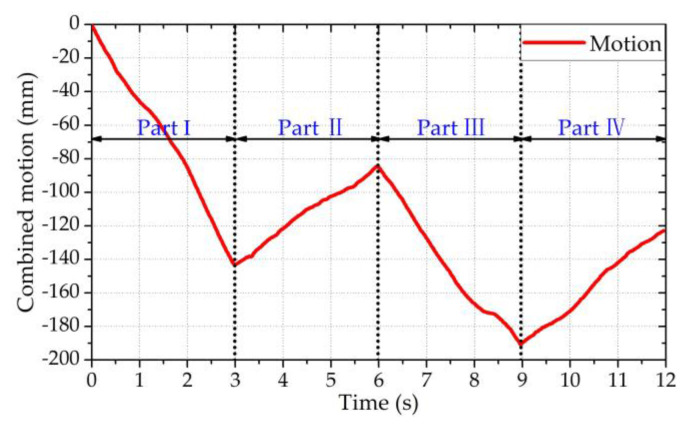
The segmented signals with a duration of 12 s.

In Figure 11, ∆*t* is equal to the reciprocal of the sampling frequency (i.e., ∆*t* = 1/*f*_s_). Figure 12 shows the combined motion signals $\hat{x}$(*n*) and the segmented signals after utilizing the segmentation algorithm.

**Figure 13:**
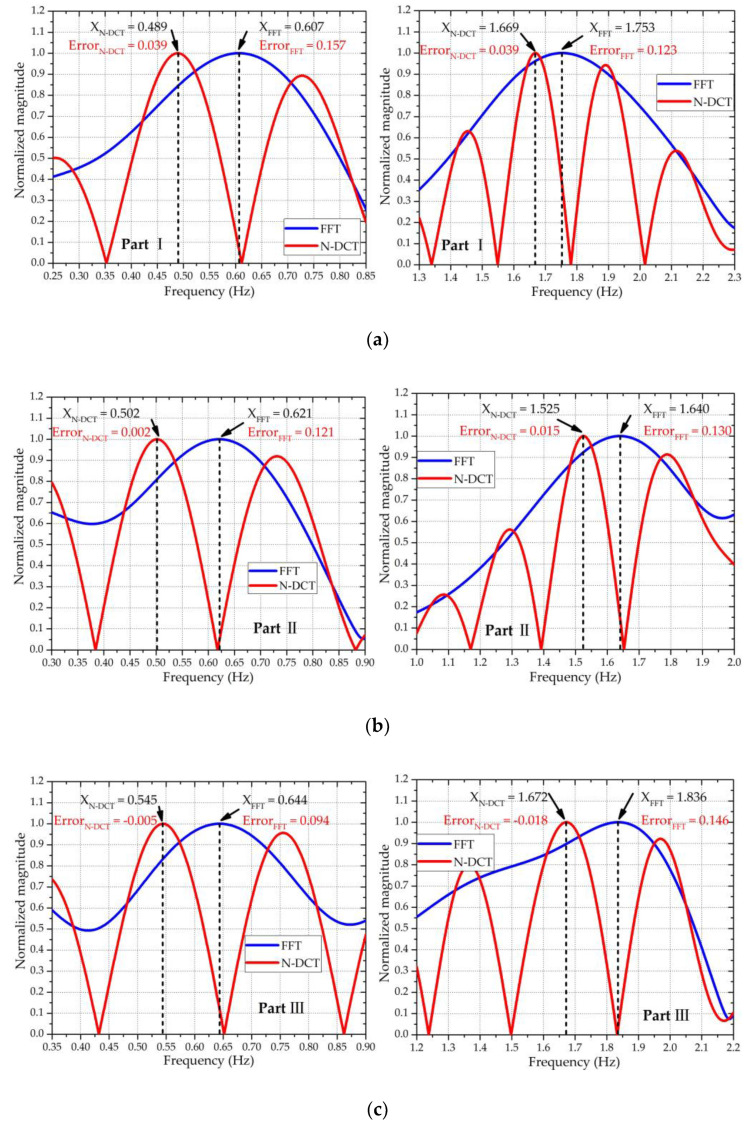

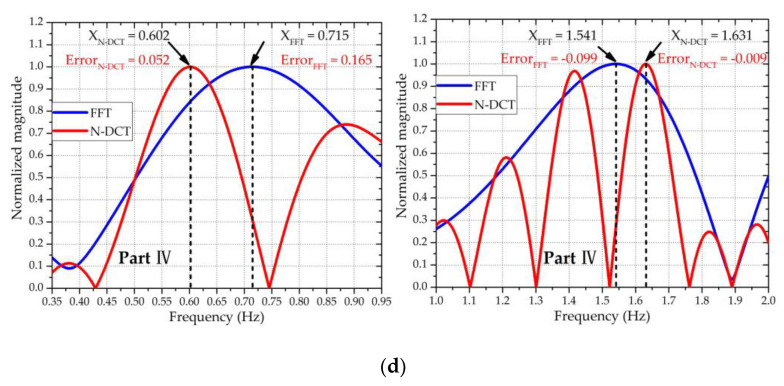
Frequency spectra of the respiration and heartbeat of the four parts corresponding to Figure 12, with the RR in the left figures and the HR in the right figures: (**a**) part Ⅰ—the true RR = 0.45 Hz, HR = 1.63 Hz; (**b**) part Ⅱ—the true RR = 0.50 Hz, HR = 1.51 Hz; (**c**) part Ⅲ—the true RR = 0.55 Hz, HR = 1.69 Hz; and (**d**) part Ⅳ—the true RR = 0.55 Hz, HR = 1.64 Hz.

The following points can be obtained from Figure 12. First, by applying the segmentation algorithm, the combined motion signals $\hat{x}$(*n*) were successfully segmented based on the body movement direction. Second, to meet the requirements of the short time window, the length of the segmented signal was about 3 s. Third, although we assumed the speed of RBM was constant during a short time window, to be as close as possible to the real-world scenario, the speed of the RBM was not always constant in a short time window. Based on the ANC method, the RR and HR were extracted using N-DCT and FFT methods with the same 3 s window width. The extracted results are shown in Figure 13.

Table 2 summarizes the measurement errors of the RR and HR with the FFT and N-DCT methods based on a 3 s time window.

As can be seen from Table 2, compared with the traditional FFT method using a 3 s time window, the N-DCT reduced the average error of the RR from 24.77% to 4.86% and reduced the average error of the HR from 7.71% to 1.25%.

Table 3 lists the comparison results with other works. It can be found that even for rigorous cases regarding longer motion ranges and faster-moving velocities, both the RR and HR can be detected with higher accuracy by utilizing the proposed scheme with only one single Doppler radar sensor.

## 5. Conclusions

In this study, to extract vital signs (i.e., the rate of respiration and heartbeat) during large-scale and fast body movements using only one single Doppler radar sensor, an ANC algorithm with the help of N-DCT was proposed. Previous studies used two Doppler radar systems or a single Doppler radar system with extra hardware to remove the body movement. However, they suffer from inconvenient usage in practical environments and have difficulties in removing large-scale or fast body movements. Further studies were done to solve these drawbacks, but they still cannot extract both the RR and HR under large-scale and fast body movements using only one single Doppler radar sensor. In response to these defects, the method proposed in this study could solve this problem. Using third-order polynomial fitting to simulate the body movement signals, the ANC algorithm was proposed to separate vital sign signals from body movement signals. Using the separated vital sign signals, the N-DCT method was introduced to extract both the RR and HR with higher accuracy. Specifically, based on a 5 s time window and compared with the traditional FFT method, utilizing the N-DCT method reduced the average error from 25.53% to 2.14% for the RR and from 3.84% to 0.87% for the HR. Meanwhile, based on a 3 s time window and compared with the traditional FFT method, utilizing the N-DCT method reduced the average error from 24.77% to 4.86% for the RR and from 7.71% to 1.25% for the HR. In addition, only general-purpose RF and communication instruments (i.e., a vector network analyzer and a spectral analyzer) were needed to build the Doppler radar sensor. For this radar sensor, no extra RF/microwave components and modules were needed, and neither was a printed circuit board nor an integrated-chip design.

In this article, we have shown the feasibility of this idea, that is, utilizing a single Doppler radar to extract the entire body motion first, then fitting the relative displacement curve of the body motion, and finally using ANC to remove the RBM and extracting vital sign signals. However, there are some limitations to this work. First, similar to References [16,17,18,19,20,21,22], the method proposed in this study could detect vital signs when only the upper body was moved randomly but not the arm. The moving arm introduced a new Doppler phase modulation, which made it more difficult to extract vital signs. The second limitation was that the speed of body movement was assumed to be constant in a short time window. In a real scenario, random body movements could be an irregular back and forth movement or a single-direction movement with speed variations. To manage a real scenario, we divided a long time window of the vital sign signal into several short time windows, where each short time window contained the body movement toward one direction with a constant speed such that the method proposed in this paper could still be used. For the worst case, during the RBM, the speed of the body movement keeps changing. To manage this case, in future work, we will adopt a more flexible fitting method to fit the motion curve such that the fitted curve is closer to the RBM curve. Then, the ANC proposed in this paper will be utilized to remove the RBM and extract vital sign signals.

## Figures and Tables

**Table 1:** Comparison of the measurement errors using an FFT and the N-DCT.

Experiment	RR		HR	
	FFT	N-DCT	FFT	N-DCT
Part Ⅰ	61.50%	−1.75%	−2.80%	0.48%
Part Ⅱ	10.17%	0.50%	−3.17%	0.42%
Part Ⅲ	13.71%	2.57%	−2.05%	0.08%
Part Ⅳ	−16.75%	−3.75%	−7.32%	−2.49%
Average	25.53%	2.14%	3.84%	0.87%

**Table 2:** Comparison of the measurement errors using an FFT and the N-DCT.

Experiment	RR		HR	
	FFT	N-DCT	FFT	N-DCT
Part Ⅰ	27.78%	8.67%	7.55%	2.39%
Part Ⅱ	24.20%	0.40%	8.61%	0.99%
Part Ⅲ	17.09%	−0.91%	8.64%	−1.07%
Part Ⅳ	30.00%	9.45%	−6.04%	−0.55%
Average	24.77%	4.86%	7.71%	1.25%

**Table 3:** Comparison of the proposed method with other existing RBM cancellation techniques.

Ref. No.	Maximum RBM Range	Maximum RBM Velocity	Maximum Detection Distance	Error of RR	Error of HR	A Single Radar Sensor	Both RR and HR Are Measured
[16]	Not Mentioned	4 mm/s	Not Mentioned	Not Mentioned	Not Mentioned	No	Yes
[17]	100 mm	Not Mentioned	1 m	Not Mentioned	Not Mentioned	No	Yes
[18]	Not Mentioned	Not Mentioned	Not Mentioned	Not Mentioned	Not Mentioned	No	Yes
[19]	60 mm	Not Mentioned	0.7 m	Not Mentioned	Not Mentioned	No	Yes
[20]	200 mm	<7.7 mm/s	1.35 m	Not Mentioned	Not Mentioned	No	Yes
[21]	150 mm	≈0 mm/s ^1^	1.5 m	Not Mentioned	Not Mentioned	Yes	Yes
[22]	Not Mentioned	20 mm/s	Not Mentioned	7.15%	Not Mentioned	Yes	Only RR
This work	155 mm	47.6 mm/s	1.5 m	2.14% ^2^	0.87% ^2^	Yes	Yes
				4.86% ^3^	1.25% ^3^		

^1^ Although the maximum RBM velocity was 50 mm/s, there needed to be a period during which the subject’s movement velocity was close to 0 mm/s to extract the vital signs. ^2^ Based on a 5 s time window. ^3^ Based on a 3 s time window.
